# Supplementary material for: Measuring Eco-Anxiety with the Polish Version of the 13-Item Hogg Eco-Anxiety Scale (HEAS-13): Latent Structure, Correlates, and Psychometric Performance
Source: Healthcare (Basel). 2024 Nov 12;12(22):2255. doi: 10.3390/healthcare12222255 (PMC11593921; doi:10.3390/healthcare12222255)
Supplement: Supplementary file 1 [file healthcare-12-02255-s001.zip › healthcare-3229522-supplementary.pdf]

Supplementary Table S1

Descriptive statistics of the HEAS-13 items and standardised factor loadings from confirmatory factor analysis (n = 634).

HEAS-13 subscales	HEAS-13 items	*M*	*SD*	Skewness	Kurtosis	Factor loadings
Affective symptoms	1. Feeling nervous, anxious or on edge.	0.70	0.78	1.15	1.25	0.63
	2. Not being able to stop or control worrying.	0.41	0.74	2.00	3.75	0.87
	3. Worrying too much.	0.32	0.68	2.39	5.49	0.84
	4. Feeling afraid.	0.47	0.65	1.42	2.25	0.69
Rumination	5. Unable to stop thinking about future climate change and other global environmental problems.	0.33	0.56	1.75	3.49	0.77
	6. Unable to stop thinking about past events related to climate change.	0.33	0.56	1.74	3.40	0.82
	7. Unable to stop thinking about losses to the environment.	0.44	0.63	1.41	1.96	0.83
Behavioural symptoms	8. Difficulty sleeping.	0.30	0.70	2.58	6.25	0.66
	9. Difficulty enjoying social situations with family and friends.	0.32	0.65	2.20	4.78	0.73
	10. Difficulty working and/or studying.	0.38	0.73	2.04	3.63	0.87
Anxiety about personal impact	11. Feeling anxious about the impact of your personal behaviours on the earth.	0.49	0.67	1.39	2.14	0.78
	12. Feeling anxious about your personal responsibility to help address environmental problems.	0.41	0.62	1.58	2.69	0.85
	13. Feeling anxious that your personal behaviours will do little to help fix the problem.	0.51	0.69	1.27	1.35	0.77

Note. The theoretically possible and empirically derived range of scores is from 0 to 3 for all HEAS-13 items.

Supplementary Table S2

The estimated correlations between the HEAS-13 subscales from confirmatory factor analysis (n = 634).

HEAS-13 subscales	Affective symptoms	Rumination	Behavioural symptoms	Anxiety about personal impact
Affective symptoms	—			
Rumination	0.45	—		
Behavioural symptoms	0.69	0.24	—	
Anxiety about personal impact	0.35	0.66	0.17	—

Note. All estimated correlations are statistically significant (all *ps* < 0.001).

Supplementary Table S3
The test-retest results of the HEAS-13 (n = 95).

Variables	*M*	*SD*	$t(94)$	*p*	Cohen's *d*	Effect size interpretation for Cohen's *d*	BF_{10}	Interpretation of BF_{10}	$ICC_{2,1}$ (95% CI)
Affective symptoms T1	0.35	0.45	3.08	0.003	0.32	Small effect	9.27	Substantial evidence for H_1	0.72 (0.59; 0.81)
Affective symptoms T2	0.25	0.39							
Rumination T1	0.34	0.56	1.68	0.097	0.17	Trivial effect	0.44	Anecdotal evidence for H_0	0.54 (0.38; 0.67)
Rumination T2	0.25	0.51							
Behavioural symptoms T1	0.19	0.44	2.39	0.019	0.25	Small effect	1.69	Anecdotal evidence for H_1	0.49 (0.32; 0.63)
Behavioural symptoms T2	0.09	0.29							
Anxiety about personal impact T1	0.55	0.67	1.31	0.193	0.13	Trivial effect	0.26	Substantial evidence for H_0	0.69 (0.57; 0.78)
Anxiety about personal impact T2	0.48	0.66							

Note. The interpretation of BF_{10} are based on the evidence categories indicated in Wetzels et al. [59].

The Polish Version of the Hogg Eco-Anxiety Scale (HEAS-13)

Skala ekolęku Hogg

Instrukcja. W ciągu ostatnich 2 tygodni, jak często dokuczały Ci następujące problemy, kiedy myślałeś/aś o zmianach klimatu i innych globalnych warunkach środowiskowych (np. o globalnym ociepleniu, degradacji środowiska, wyczerpywaniu się zasobów, wymieraniu gatunków, dziurze ozonowej, zanieczyszczeniu oceanów, wylesianiu)?

		Wcale	Przez kilka dni	Przez więcej niż połowę dni	Prawie każdego dnia
1	Zdenerwowanie, lęk lub irytacja.	0	1	2	3
2	Trudności związane z opanowaniem zamartwiania się.	0	1	2	3
3	Zbyt silne zamartwianie się.	0	1	2	3
4	Odczuwanie strachu.	0	1	2	3
5	Niemożność zaprzestania myślenia o przyszłych zmianach klimatu i innych globalnych problemach środowiskowych.	0	1	2	3
6	Niemożność zaprzestania myślenia o przeszłych wydarzeniach związanych ze zmianami klimatu.	0	1	2	3
7	Niemożność zaprzestania myślenia o stratach środowiskowych.	0	1	2	3
8	Trudności ze snem.	0	1	2	3
9	Trudności w cieszeniu się sytuacjami społecznymi z rodziną i przyjaciółmi.	0	1	2	3
10	Trudności w pracy (pracowaniu) i/lub uczeniu się.	0	1	2	3
11	Odczuwanie niepokoju z powodu wpływu własnych zachowań na Ziemię.	0	1	2	3
12	Odczuwanie niepokoju związanego z własną odpowiedzialnością za pomoc w rozwiązywaniu problemów środowiskowych.	0	1	2	3
13	Odczuwanie niepokoju z powodu tego, że własne zachowania w małym stopniu przyczynią się do rozwiązania problemu.	0	1	2	3

Skala ekolęku Hogg (*Hogg Eco-Anxiety Scale*; HEAS-13) jest 13-pozycyjnym samoopisowym kwestionariuszem do badania ekoleku (lęku klimatycznego). Składa się z czterech podskal: (1) objawy afektywne (*affective symptoms*), (2) ruminacja (*rumination*), (3) objawy behawioralne (*behavioural symptoms*), (4) niepokój związany z własnym oddziaływaniem (*anxiety about personal impact*).

Poniższa tabela opisuje podskale HEAS-13 oraz sposób ich obliczania. Wyższe wyniki w tych podskalach oznaczają wyższą częstotliwość doświadczania objawów ekolęku.

Podskale	Jak obliczyć?	Co mierzy?
Objawy afektywne	Średni wynik sumy pozycji 1, 2, 3, 4.	Symptomy lęku związane ze zmianami klimatu.
Ruminacja	Średni wynik sumy pozycji 5, 6, 7.	Ciągłe myślenie o zmianach klimatu.
Objawy behawioralne	Średni wynik sumy pozycji 8, 9, 10.	Objawy behawioralne związane z reakcją na zmiany klimatu.
Niepokój związany z własnym oddziaływaniem	Średni wynik sumy pozycji 11, 12, 13.	Objawy lęku związane z własnym oddziaływaniem/wpływem na kwestie środowiskowe.
